# COVID-19 e Fibrilação Atrial: Prevendo para Prevenir

**DOI:** 10.36660/abc.20230823

**Published:** 2024-03-20

**Authors:** Lucas Simonetto Faganello, Leandro Ioschpe Zimerman

**Affiliations:** 1 Hospital de Clínicas de Porto Alegre Porto Alegre RS Brasil Hospital de Clínicas de Porto Alegre, Porto Alegre, RS – Brasil

**Keywords:** Infecções por Coronavírus, Fibrilação Atrial/mortalidade, Doença da Arterial Coronariana, Síndrome Respiratória Aguda Grave

A fibrilação atrial (FA) é a arritmia crônica mais comum em todo o mundo, com significativa morbidade e mortalidade.^1^ As causas para desenvolvimento e manutenção da FA são multifatoriais, estando sob maior risco os pacientes com idade avançada e comorbidades como hipertensão, diabetes e doença arterial coronariana. Aumento do tônus simpático e estados inflamatórios também estão associados ao desenvolvimento de FA.^1 , 2^

A pandemia da doença pelo novo coronavírus que se iniciou no final de 2019 (COVID-19) rapidamente se alastrou pelo mundo trazendo consequências catastróficas para saúde pública e economia global.^3^ A infecção pode levar a doença com severidades variadas, variando desde infecção leve de via aérea alta até a pneumonia grave, resultando em síndrome respiratória aguda grave com necessidade de ventilação mecânica. Embora a COVID-19 seja primariamente uma doença pulmonar como consequência de uma resposta inflamatória imune, o envolvimento cardiovascular pode compor o quadro.^3 , 4^ Manifestações cardíacas incluem miocardite, insuficiência cardíaca, síndrome coronariana aguda, arritmias, e estão associadas a maior mortalidade. O mecanismo arritmogênico exato ainda não é completamente conhecido, mas hipoxia, miocardite, resposta imune exagerada, isquemia miocárdica, distúrbio eletrolítico e efeitos adversos de medicações são potenciais causas.^4 , 5^

Incidência elevada de FA tem sido documentada em pacientes com COVID-19, estando associada com piores desfechos clínicos.^6^ Segundo uma pesquisa online realizada pela *Heart Rhythm Society* em 2020 com 1.197 respondedores de 76 países diferentes, a FA foi a arritmia mais reportada, presente em 20% dos pacientes internados por COVID-19.^7^ Pimentel et al.,^8^ por sua vez, evidenciaram que a presença de arritmias em 241 pacientes consecutivos internados com COVID-19 em um hospital brasileiro foi de 8,7%, sendo 76,2% arritmias atriais. A presença de insuficiência cardíaca foi a única variável associada a maior risco de arritmia cardíacas em análise multivariada.^8^ Em uma pesquisa mundial publicada pelo *Circulation* em 2021, com a participação de 12 países, incluindo o Brasil, a maioria dos pacientes que desenvolveram alguma arritmia durante a internação por COVID-19, não tinham história de arritmia.^9^ Dos pacientes que apresentaram arritmia, a FA foi relatada em 61% dos casos. De maneira similar ao descritos em estudos prévios, a presença de arritmia se associou com significativa morbimortalidade; 43% dos pacientes que desenvolveram arritmia foram submetidos a ventilação mecânica, sendo que somente 51% sobreviveram até a alta hospitalar. Bernstein et al.,^10^ por sua vez, analisaram a incidência de FA através de uma coorte multicêntrica retrospectiva com 39.415 pacientes internados com diagnóstico de pneumonia e sem diagnóstico prévio de FA. A incidência em pacientes com pneumonia por COVID foi menor quando comparada a pacientes com pneumonia não COVID (10% versus 14%). Os valores de troponina foram normais, sugerindo que a ocorrência de FA esteja relacionada com a severidade da doença de base, e não propriamente com injúria miocárdica.^10^

Nesta edição dos Arquivos Brasileiros de Cardiologia, Andrea et al.,^11^ acrescentam informações relevantes ao tema. Em um estudo caso-controle com 201 pacientes admitidos com o diagnóstico de pneumonia viral por COVID-19, 14,4% apresentaram FA durante a internação. Estes tinham idade mais avançada, maior prevalência de doenças neurológicas crônicas e maior número de comorbidades, associando-se a um contexto de maior gravidade durante a hospitalização. A internação em Unidade de Terapia Intensiva, e a necessidade de ventilação mecânica associaram-se à ocorrência de FA. Análise multivariada demonstrou que idade > 71 anos, leucometria ≤ 7.720 cels.μL^-^¹, natremia ≤ 137 mEq.L^-^¹, SAPS 3 ( *Simplilfied Acute Physiology Score)* > 55 e desorientação foram preditores independentes para ocorrência de FA durante a internação. Os autores foram além, através de uma modelagem prognóstica para a ocorrência de FA. Foi desenvolvido um sistema de pontuação, em que para cada variável do modelo foi atribuído o valor unitário quando ela se encontrava na faixa de gravidade, ou zero quando fora da faixa. Utilizando a curva ROC, o valor de corte ótimo do escore de gravidade para FA > 2 apresentou uma especificidade de 65,2% e uma sensibilidade de 82,8%.

O verdadeiro impacto da pandemia ainda está para ser definido. A real incidência de miocardite, fibrose miocárdica, episódios de FA assintomáticos e sintomáticos, e suas consequências, são lacunas a serem elucidadas.^12 - 15^ A ocorrência de FA em pacientes com COVID-19 parece estar relacionada à gravidade da apresentação clínica e laboratorial, não havendo evidência inequívoca de que a infecção por esse patógeno seja mais arritmogênica quando comparada a outras infecções virais.^10 , 16^ Entretanto, por se tratar de uma doença com atividade pró trombótica, uma análise objetiva do risco de desenvolver uma arritmia como a FA pode acrescentar informações valiosas no manejo desses pacientes. Prever talvez possa ser a melhor prevenção, na busca de reduzir sintomas e riscos de eventos tromboembólicos. A produção científica contínua, baseada em dados atualizados é fundamental para a construção da melhor estratégia terapêutica.
