# Supplementary material for: COVID-19 e Fibrilação Atrial: Prevendo para Prevenir
Source: Arq Bras Cardiol. 2024 Mar 20;121(1):e20230823. [Article in Portuguese] doi: 10.36660/abc.20230823 (PMC11081095; doi:10.36660/abc.20230823)
Supplement: Translation

# Translation: COVID-19 and Atrial Fibrillation: Predicting to Prevent

**Authors:** Lucas Simonetto Faganello, Leandro Ioschpe Zimerman

**Affiliations:** 1 Porto Alegre RS Brazil Hospital de Clínicas de Porto Alegre, Porto Alegre, RS – Brazil

**Keywords:** Coronavirus Infections, Atrial Fibrillation/mortality, Coronary Artery Disease, Severe Acute Respiratory Syndrome

Atrial fibrillation (AF) is the most common chronic arrhythmia worldwide, with significant morbidity and mortality.^1^The causes for the development and maintenance of AF are multifactorial, with advanced-age patients and comorbidities such as hypertension, diabetes, and coronary artery disease being at greater risk. Increased sympathetic tone and inflammatory states are also associated with the development of AF.^1 , 2^

The new coronavirus disease pandemic that began at the end of 2019 (COVID-19) quickly spread across the world, bringing catastrophic consequences for public health and the global economy.^3^The infection can lead to illness with varying severities, ranging from mild upper airway infection to severe pneumonia, resulting in severe acute respiratory syndrome requiring mechanical ventilation. Although COVID-19 is primarily a lung disease as a consequence of an immune inflammatory response, cardiovascular system may be involved as well.^3 , 4^Cardiac manifestations include myocarditis, heart failure, acute coronary syndrome, and arrhythmias and are associated with increased mortality. The exact arrhythmogenic mechanism is not yet completely known, but hypoxia, myocarditis, exaggerated immune response, myocardial ischemia, electrolyte disturbance, and adverse effects of medications are potential causes.^4 , 5^

A high incidence of AF has been documented in patients with COVID-19, being associated with worse clinical outcomes.^6^According to an online survey carried out by the Heart Rhythm Society in 2020 with 1,197 respondents from 76 different countries, AF was the most reported arrhythmia, present in 20% of patients hospitalized for COVID-19.^7^Pimentel et al.,^8^showed that the presence of arrhythmias in 241 consecutive patients admitted with COVID-19 in a Brazilian hospital was 8.7%, 76.2% of which were atrial arrhythmias. The presence of heart failure was the only variable associated with a higher risk of cardiac arrhythmias in multivariate analysis.^8^In a global survey published by Circulation in 2021, with the participation of 12 countries, including Brazil, the majority of patients who developed an arrhythmia during hospitalization for COVID-19 had no history of arrhythmia.^9^Of the patients who presented with arrhythmia, AF was reported in 61% of cases. Similar to what was described in previous studies, the presence of arrhythmia was associated with significant morbidity and mortality; 43% of patients who developed arrhythmia were subjected to mechanical ventilation, with only 51% surviving until hospital discharge. Bernstein et al.,^10^analyzed the incidence of AF through a retrospective multicenter cohort with 39,415 patients hospitalized with a diagnosis of pneumonia and without a previous diagnosis of AF. The incidence in patients with COVID pneumonia was lower when compared to patients with non-COVID pneumonia (10% versus 14%). Troponin values were normal, suggesting that the occurrence of AF is related to the severity of the underlying disease and not exactly to myocardial injury.^10^

In this edition of the *Arquivos Brasileiros de Cardiologia* , Andrea et al.,^11^add relevant information to the topic. In a case-control study with 201 patients admitted with a diagnosis of viral pneumonia due to COVID-19, 14.4% had AF during hospitalization. These had older age, higher prevalence of chronic neurological diseases, and a greater number of comorbidities associated with a context of greater severity during hospitalization. Admission to the Intensive Care Unit and the need for mechanical ventilation were associated with the occurrence of AF. Multivariate analysis demonstrated that age > 71 years, leukometry ≤ 7,720 cells.μL-¹, natremia ≤ 137mEq.L-¹, SAPS 3 (Simplified Acute Physiology Score) > 55, and disorientation were independent predictors for the occurrence of AF during hospitalization. The authors went further through prognostic modeling for the occurrence of AF. A scoring system was developed in which each variable in the model was assigned a value of unity when it was within the severity range or zero when outside the range. Using the ROC curve, the optimal cutoff value of the severity score for AF > 2 had a specificity of 65.2% and a sensitivity of 82.8%.

The true impact of the pandemic is yet to be defined. The real incidence of myocarditis, myocardial fibrosis, asymptomatic and symptomatic episodes of AF, and their consequences are gaps to be elucidated.^12 - 15^The occurrence of AF in patients with COVID-19 appears to be related to the severity of the clinical and laboratory presentation, and there is no unequivocal evidence that infection with this pathogen is more arrhythmogenic when compared to other viral infections.^10 , 16^However, as it is a disease with prothrombotic activity, an objective analysis of the risk of developing an arrhythmia such as AF can add valuable information to the management of these patients. Forecasting could perhaps be the best prevention in the quest to reduce symptoms and risks of thromboembolic events. Continuous scientific production based on updated data, is essential for building the best therapeutic strategy.
